# Advancing the scholarship and practice of stakeholder engagement in working landscapes: a co-produced research agenda

**DOI:** 10.1007/s42532-022-00132-8

**Published:** 2022-11-11

**Authors:** Weston M. Eaton, Morey Burnham, Tahnee Robertson, J. G. Arbuckle, Kathryn J. Brasier, Mark E. Burbach, Sarah P. Church, Georgia Hart-Fredeluces, Douglas Jackson-Smith, Grace Wildermuth, Katherine N. Canfield, S. Carolina Córdova, Casey D. Chatelain, Lara B. Fowler, Mennatullah Mohamed Zein elAbdeen Hendawy, Christine J. Kirchhoff, Marisa K. Manheim, Rubén O. Martinez, Anne Mook, Cristina A. Mullin, A. Laurie Murrah-Hanson, Christiana O. Onabola, Lauren E. Parker, Elizabeth A. Redd, Chelsea Schelly, Michael L. Schoon, W. Adam Sigler, Emily Smit, Tiff van Huysen, Michelle R. Worosz, Carrie Eberly, Andi Rogers

**Affiliations:** 1 University of Wyoming, Laramie, USA; 2 Idaho State University, Pocatello, ID, USA; 3 Southwest Decision Resources, Tucson, USA; 4 Iowa State University, Ames, USA; 5 Penn State University, State College, USA; 6 University of Nebraska-Lincoln, Lincoln, USA; 7 Montana State University, Bozeman, MT, USA; 8 Ohio State University, Columbus, USA; 9 U.S. Environmental Protection Agency, Washington, USA; 10 Barnstable Clean Water Coalition, Osterville, USA; 11 Ain Shams University, Cairo, Egypt; 12 School of Sustainability, Arizona State University, Arizona, USA; 13 Michigan State University, East Lansing, USA; 14 Colorado State University, Fort Collins, USA; 15 Independent Researcher, Salt Lake City, USA; 16 University of Northern British Columbia, Prince George, Canada; 17 USDA California Climate Hub, Washington, USA; 18 Michigan Technological University, Houghton, MI, USA; 19 Arizona State University, Arizona, USA; 20 University of Toronto, Toronto, Canada; 21 USDA Agricultural Research Service, Maryland, USA; 22 Alabama Agricultural Experiment Station, Auburn University, Auburn, USA

**Keywords:** Community and stakeholder engagement, Working lands, Research-practice gaps, Process design, Knowledge co-production, Engagement outcomes

## Abstract

Participatory approaches to science and decision making, including stakeholder engagement, are increasingly common for managing complex socio-ecological challenges in working landscapes. However, critical questions about stakeholder engagement in this space remain. These include normative, political, and ethical questions concerning who participates, who benefits and loses, what good can be accomplished, and for what, whom, and by who. First, opportunities for addressing justice, equity, diversity, and inclusion interests through engagement, while implied in key conceptual frameworks, remain underexplored in scholarly work and collaborative practice alike. A second line of inquiry relates to research–practice gaps. While both the practice of doing engagement work and scholarly research on the efficacy of engagement is on the rise, there is little concerted interplay among ‘on-the-ground’ practitioners and scholarly researchers. This means scientific research often misses or ignores insight grounded in practical and experiential knowledge, while practitioners are disconnected from potentially useful scientific research on stakeholder engagement. A third set of questions concerns gaps in empirical understanding of the efficacy of engagement processes and includes inquiry into how different engagement contexts and process features affect a range of behavioral, cognitive, and decision-making outcomes. Because of these gaps, a cohesive and actionable research agenda for stakeholder engagement research and practice in working landscapes remains elusive. In this review article, we present a co-produced research agenda for stakeholder engagement in working landscapes. The co-production process involved professionally facilitated and iterative dialogue among a diverse and international group of over 160 scholars and practitioners through a yearlong virtual workshop series. The resulting research agenda is organized under six cross-cutting themes: (1) Justice, Equity, Diversity, and Inclusion; (2) Ethics; (3) Research and Practice; (4) Context; (5) Process; and (6) Outcomes and Measurement. This research agenda identifies critical research needs and opportunities relevant for researchers, practitioners, and policymakers alike. We argue that addressing these research opportunities is necessary to advance knowledge and practice of stakeholder engagement and to support more just and effective engagement processes in working landscapes.

## 1 The need for a renewed research agenda for stakeholder engagement

Complex socio-ecological problems threaten food production, human health, and ecological integrity. These problems are especially consequential for working landscapes, defined as spaces where livelihood is inextricably linked with policy and the use and management of land, water, and other natural resources (Plieninger et al. 2012, p. 428). To improve socio-ecological outcomes through research and policy, researchers, practitioners, and policymakers increasingly turn to collaborative and participatory approaches, including stakeholder engagement (Newig et al. 2018; Jager et al. 2020; Feist et al. 2020). Engagement refers to processes where stakeholders are involved in making decisions that affect them (Eaton et al. 2021, p. 1113). We define stakeholders broadly as individuals or groups that can affect or will be affected by research and policy decisions, solutions, and actions (Reed et al. 2018, p. 2). The social interaction among diverse stakeholders at the core of the engagement process is believed to foster learning, support coordination, and build the shared visions and practical capacities needed for improving socio-ecological outcomes (Berkes 2009; Pahl-Wostl 2009; Muro and Jeffrey 2012; Gerlak et al. 2018). However, while various forms of stakeholder engagement are widely used, evidence for their effectiveness for addressing and solving complex socio-ecological problems is currently lacking (Gerlak et al. 2019, p. 2; Feist et al. 2020, p. 802).

Advancing stakeholder engagement research and practice requires addressing several pressing knowledge gaps. First are normative, political, and ethical questions about who benefits, who loses, and what good can be accomplished for and by whom through engagement. This line of inquiry follows Arnstein’s (1969) call for participatory approaches that aim to empower less powerful actors to join in co-making decisions in ways that generate social reform geared toward creating a more socially and ecologically equitable and just society (Cook and Zurita 2019, p. 57). Moreover, asking such questions extends critical perspectives on collaborative governance that examine how failure to convene inclusive, democratic processes tend to heighten distrust and sustain unethical, unjust, and unsustainable environmental practices (Cleaver 2001; Bluhdorn and Deflorian 2019). Knowledge—both practical and scientific—on whether and how stakeholder engagement addresses justice, equity, diversity, and inclusivity (JEDI) concerns, remains nascent (Som Castellano and Mook 2022). While JEDI is woven throughout classic and contemporary conceptual frameworks for doing and researching engagement (Arnstein 1969; Ansell and Gash 2008; Emerson et al. 2012), more work examining the intersection of JEDI with stakeholder engagement in working landscapes is needed (Dobbin and Lubell 2021).

Second, while practitioners and scholars have identified numerous best practices for stakeholder engagement in environmental contexts (e.g., Schusler et al. 2003, p. 317–322; Kliskey et al. 2021), questions remain pertaining to roles for and relationships among ‘on-the-ground’ practitioners convening and facilitating engagement processes; researchers and practitioners employing, designing, studying, and evaluating those processes and outcomes; and community stakeholders participating in engagement processes. Central here is identifying and overcoming barriers for marrying practical and experiential knowledge, as well as traditional and Indigenous knowledge systems, with scientific knowledge. Few venues exist for interdisciplinary conversations joining practical, experiential, Indigenous, and scientific knowledge among individuals who design, convene, facilitate, study, support, participate with, or perform various combinations of these and related roles regarding stakeholder engagement. As a starting point, building new connections among stakeholder engagement researchers and practitioners can catalyze research that better supports engagement processes and outcomes, and better integrates practical and experiential insight with theoretical understanding.

Third, scholars, practitioners, and policy experts alike seek evidence for the efficacy of stakeholder engagement, including how engagement processes and the context within which they unfold yield either positive or negative policy and socio-ecological outcomes (Koontz and Thomas 2006; Newig et al. 2018; Gerlak et al. 2018). The current lack of evidence for the effectiveness of engagement prompt the need for critical reflection on the worth and risk of convening diverse stakeholders to revisit status quo approaches to undertaking and governing research, planning, and policy decision making (Innes and Booher 1999; Lukasiewicz and Baldwin 2017). Without improved knowledge of processes and outcomes of stakeholder engagement, and the ethical dimensions of contexts within which they unfold, we risk wasting resources, failing to anticipate unintended consequences, damaging relationships, and ultimately failing to achieve lasting socio-ecological transformation (Bluhdorn and Delflorian 2019; Huang and Harvey 2021, p. 1–3).

In this paper, we present a research agenda co-produced through a workshop series that joined over 160 diverse researchers and practitioners whose work is focused on stakeholder engagement in working landscapes in varying capacities in a yearlong facilitated process. These workshops catalyzed the papers collected in this special issue, as well as identified 34 research opportunities organized into the six cross-cutting themes presented in this paper. We next describe our knowledge co-production process and workshop participants. We then detail each of the six themes and related research opportunities and conclude with an invitation for participation in next steps.

## 2 Advancing engagement workshop series

### 2.1 Process, participants and methods

To address questions on the efficacy of stakeholder engagement, the workshop organizing team (consisting of researchers and practitioners from Penn State University, Idaho State University, Iowa State University, The Ohio State University, University of Nebraska-Lincoln, Montana State University, and Southwest Decision Resources) convened a new international community of researchers and practitioners to review current knowledge and co-produce new understandings of knowledge gaps and needs related to stakeholder engagement practice and research through a series of interactive, virtual, and professionally facilitated workshops. The ‘Advancing Engagement Workshop Series’ consisted of four workshops and additional formal and informal interactions convened between October 2020 and October 2021.^1^ These workshops enabled researchers and practitioners from diverse disciplines, backgrounds, geographies, and career stages to compare research methods and results from their work examining the effectiveness of stakeholder engagement, and collaboratively assess the current state of knowledge within research and practice of stakeholder engagement on working landscapes and discuss the key gaps in our collective understanding of stakeholder engagement. The virtual format enabled the participation of numerous researchers and practitioners around the world.

Our workshop goals included: (1) building a new diverse and international network of researchers and practitioners with a shared interest in stakeholder engagement, (2) supporting the development of new scholarship that addresses pressing knowledge gaps in stakeholder engagement practice and research (including this special issue), and (3) co-producing a future research agenda around stakeholder engagement in working landscapes.^2^ This paper focuses on the outcomes of the third goal.

### 2.2 Workshop participants

To convene a new international and multidisciplinary network, the organizing team distributed a call for participation (https://engagementworkshop2021.wordpress.com/) that included proposed workshop themes via professional networks, including topically relevant professional society listservs. The organizing team also sought participation beyond university researchers by sharing the announcement with practitioner networks and funding agencies (e.g., United States Department of Agriculture), and encouraged recipients to share the announcement internationally through their relevant networks.^3^ To apply, prospective participants were asked to complete an initial online (Qualtrics) survey that collected contact information, institutional affiliation, short biographies, and a description of how they expected to benefit from participation and how their participation would benefit others. This survey was active throughout the workshop series and received over 160 total completed responses. All individuals who completed this initial survey were invited to participate in the first workshop (additional participants were welcomed to join throughout the process). Biographies revealed individuals were affiliated with private, government, and academic institutions. Expected benefits included learning with others, sharing applied and scholarly knowledge, networking, and identifying new collaborators. Overall, participants—about half of whom identified as early career—wore multiple professional hats and identities and represented diverse professional, cultural, and ethnic backgrounds and interests related to stakeholder engagement:

- Academic faculty from research and teaching universities primarily in the USA, but also including institutions based in Australia, Bangladesh, Canada, Egypt, India, Kazakhstan, Mexico, the UK, the European Union, and central European countries.
- Graduate students, postdoctoral scholars, and other early career researchers and practitioners.
- Practitioners and professionals with government agencies primarily based in the USA, but also Canada, including individuals employed by the U.S. Environmental Protection Agency, the U.S. Forest Service, and U.S. Department of Agriculture (USDA).
- Practitioners and professionals with non-governmental organizations focused on wildlife and natural resource conservation including American Farmland Trust, National Wildlife Federation, Practical Farmers of Iowa, and Soil and Water Conservation Society.
- Professionals, researchers, and educators with state Cooperative Extension organizations.
- Professionals affiliated with Indigenous Nations and organizations.
- Researchers with USDA Agricultural Research Service.
- Professionals with agricultural experiment stations.
- Professionals with private stakeholder engagement consulting firms.

Participants were asked to complete two additional online surveys (Qualtrics, Idaho State University IRB # FY 2021-29) prior to the kickoff workshops in October 2020 to inform workshop design. The first (n = 118) inquired into gender (64% female/she/her), racial/ethic identities of participants (20% non-white), as well as localities in which participants do engagement work (33 total countries; 49 U.S. states plus Puerto Rico and Pacific Islands), engagement settings (ranging from rural to urban, public to private), research/project topics (over 50, ranging from sustainability to waste management), who they engage (top five: NGOs, university researchers, Extension, ranchers, and coalitions/collaboratives) and aspire to engage (top five: Tribal groups, migrants, youth, federal government, and local businesses). The survey also asked how participants characterize their engagement work (e.g., research, practice). Findings show experience among the group weighted toward research, although about two-thirds of participants also report spending at least some of their time facilitating engagement, as shown in Table 1. See Online Appendix A data for more detailed survey results. A second survey informed the research agenda co-production process as reported in the next section.

In all, over 160 individuals participated in at least one virtual workshop event throughout the yearlong process. We describe each event in more detail below. Participants told us time constraints were a common attrition factor. A handful of individuals that left early on shared that the project did not meet their expectations.

### 2.3 Workshop series to co-produce a research agenda

Along with building a new collaborative research network and generating content for a special issue journal, workshop series participants were asked to help co-produce a research agenda that identified pressing research gaps and opportunities with the goal of advancing a shared knowledge base for stakeholder engagement. Co-produced knowledge joins scientific and technical knowledge with practical, traditional, local, experiential, and other ways of knowing (Eden et al. 2016; Kirchhoff et al. 2013). Co-production is critical in the space of stakeholder engagement as neither practical insight nor scholarly knowledge alone is sufficient for advancing understanding for how and the circumstances by which stakeholder engagement succeeds or fails in addressing socio-ecological challenges. Our knowledge co-production process (detailed below) aimed to integrate *practical* and *experiential* knowledge with *scientific* knowledge about stakeholder engagement research and methods to develop a research agenda useful for bettering practice and research alike. We designed the knowledge co-production process to provide opportunity, in egalitarian and iterative fashion, for listening to and sharing perspectives on the status of stakeholder engagement research and practice to build a shared understanding among the network for practical needs and knowledge gaps. We believe this allowed for a greater examination of the state of knowledge and research needs and opportunities than reviewing only published research or incorporating only practical insight.

**Fig. 1:**
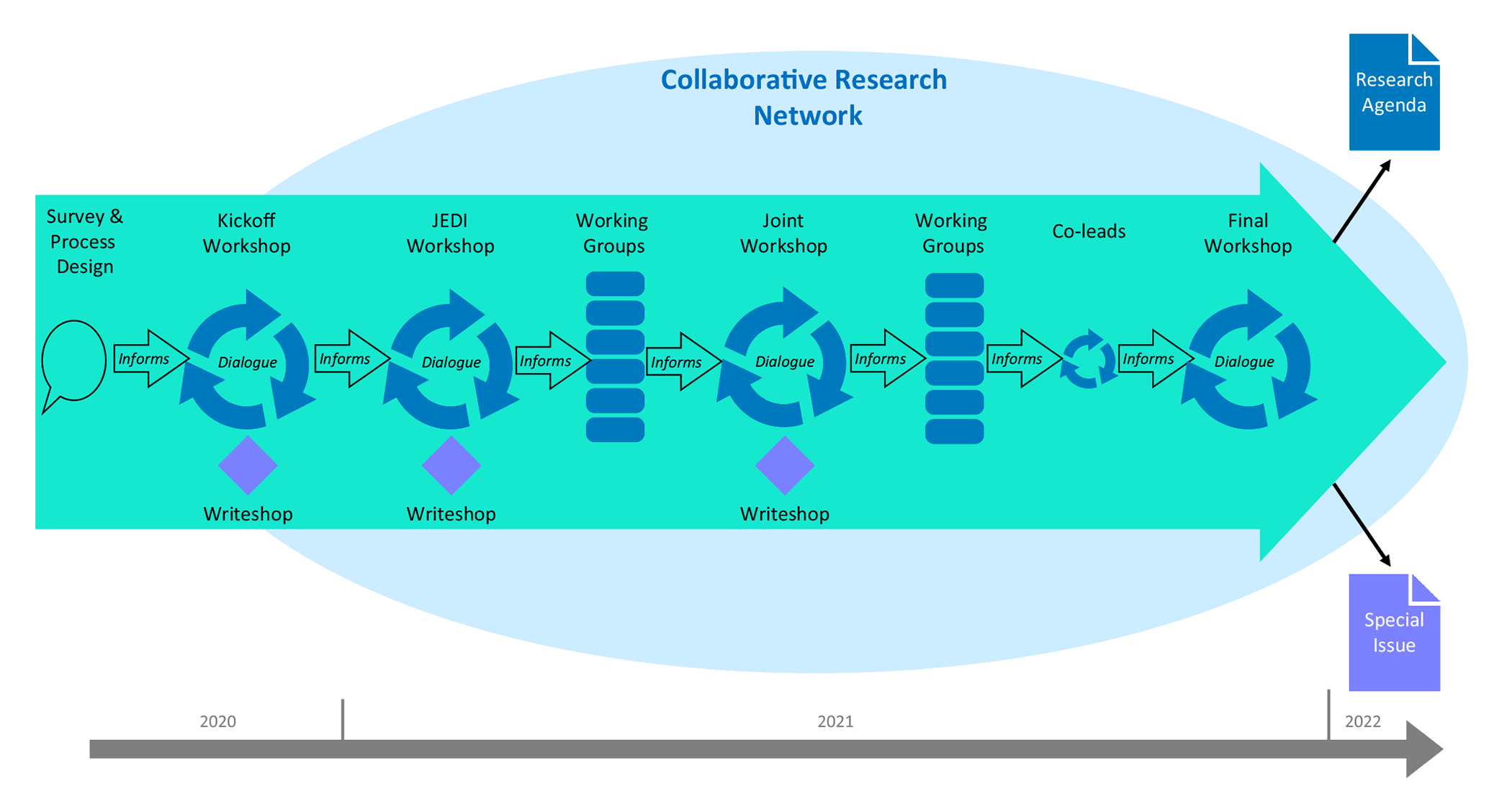
Engagement workshop series co-production process

The organizing team had originally planned to hold an in-person workshop, but COVID-19 forced transitioning to a virtual format. This switch fortuitously opened participation geographically and offered a more robust, iterative process over time. The organizing team began meeting during Summer 2020 to design a collaborative process to engage workshop participants using zoom video conferencing (see Fig. 1). Core components of this design process included an initial participant survey, a series of workshops, working group activities, and related interactions. The organizing team prepared extensive notes from each activity which were shared back with all participants throughout the process.

#### 2.3.1 Step 1: Pre-workshop research agenda survey (October 1–23, 2020)

An open-ended survey (n = 102) encouraged participants to identify up to three knowledge gaps with the following prompts: Thinking about your experience in the field of stakeholder engagement:

- What do you think are the main knowledge gaps in the science of engagement?
- Why do you think these are important?

The survey generated 238 responses. The organizing team grouped responses into six initial thematic areas, each with several subpoints, used to structure future workshop activities.

#### 2.3.2 Step 2: Kickoff workshop (October 20 & 23, 2020)

The first workshop (n = 123) facilitated small group breakout sessions to further develop the research agenda. The initial session focused on ‘what researchers want to know from practitioners’ and vice versa. The next session prompted breakout group members to identify key features of a successful research agenda, which led to this list: ^4^

- Common, accessible language
- Shared goals among a growing network of researchers, practitioners, and stakeholders
- Timeline and process with clear goals and products
- A process for continual refinement overtime

The final workshop session asked breakout group members to review each of the six thematic research gap areas derived from participant surveys and then discuss these in relation to four prompts:

- General thoughts?
- What resonates with you and why?
- What surprises you and why?
- What’s missing?

Following the workshop, three organizing team members coded breakout group responses in two steps. First, they compared new workshop content with the initial six thematic areas. Once this was completed, coders shared their work with the rest of the team, discussed ways the new content confirmed or raised questions about preliminary thematic areas, and came to agreement on a revised list of six thematic areas (see below) that were used to organize future workshop activities. Workshop organizers also produced detailed summaries of each breakout session and shared them with all participants.

1. Who participates in engagement activities, and who does not?
2. What are pressing ethical questions for engagement?
3. How are engagement research and practice integrated, if at all?
4. What shapes modes and processes of engagement?
5. How do different modes and processes of engagement affect outcomes?
6. What are the outcomes of engagement? How do we measure them?

#### 2.3.3 Step 3: Justice, equity, diversity and inclusion (JEDI) workshop (February 16, 2021)

JEDI emerged as a critical topic for our network to purposefully and intentionally address. In response, the organizing team chose to create a JEDI focused workshop. This second virtual workshop (n > 80) featured a panel and follow-up facilitated discussion on the nexus of JEDI and the emerging research agenda.

Workshop participants then self-selected into focused working groups to address the six questions listed above. Working groups were charged with developing ten-page whitepapers over the remainder of the workshop series. These whitepapers aimed to 1) briefly summarize key research and practice knowledge related to their theme, 2) identify key research gaps, and 3) identify specific research questions that should be taken up in future research. Finally, each group identified two or more ‘co-leads’ charged with facilitating working group dialogue and interaction, making decisions about how tasks would be completed, and reporting back during formal workshop activities.

#### 2.3.4 Step 4: Separate working group meetings (March 2021)

Working groups met independently to review their research agenda topics, develop their state of the literature summary, identify knowledge gaps, and collectively identify research opportunities to address those gaps. Organizers encouraged co-leads to approach the tasks in whichever way seemed most effective to them. For example, some groups convened their working meetings around a shared conceptual graphic to guide discussion and identify key outputs. Other groups collectively compiled written literature reviews using a shared document as a basis for their identification of gaps and research needs. Still others held meetings where members were responsible for sharing perspectives and, together, compiling notes in iterative fashion. Regardless of approach, these workgroup conversations served as a springboard for the joint working group workshop.

#### 2.3.5 Step 5: Joint working group workshop (April 2021)

During this workshop (n > 80), working groups met in facilitated concurrent breakout sessions to further develop their whitepapers, discuss how each individual member was contributing, identify tasks and timeframes, and report on progress to all working groups. The workshop concluded with working groups meeting together to share their experience and perspectives with other groups. This process identified shared themes and clarified the division of labor across the workgroups.

#### 2.3.6 Step 6: Working group co-lead meetings (May 2021)

Working group co-leads next met together with the organizing team to assess progress and discuss areas of help needed to complete whitepaper development.

#### 2.3.7 Step 7: Final workshop (June 9 & 10, 2021)

The final organized workshop (n > 70) included three activities. First, working groups met in concurrent breakout sessions to solidify their list of priority research opportunities for their respective working group thematic area. Next, workshop participants together identified areas of convergence found within the six working group themes and related research opportunities. Finally, participants reexamined three thematic areas (context, process, and outcomes) through the lenses of JEDI and ethics.

#### 2.3.8 Step 8: Working group co-lead meetings (July–November 2021)

Working group co-leads continued to meet to assess synergies and combine each group’s top research priorities into a comprehensive research agenda.

This iterative and facilitated co-production process resulted in the identification of 34 priority research opportunities grouped into the six cross-cutting thematic areas detailed below. The order presented does not reflect the ranking of themes and overlap may exist:

1. Justice, Equity, Diversity, and Inclusion
2. Ethics
3. Research and Practice
4. Context
5. Process
6. Outcomes and Measurement

## 3 Research agenda: thematic overview and research needs

### 3.1 Theme 1: justice, equity, diversity, and inclusion

Both the historical and present conditions of injustice, inequity, and exclusion shape contemporary engagement processes and their outcomes. Although JEDI issues are relevant in all engagement processes, we addressed these issues through an explicit working group process focused specifically on these themes. Engagement processes always involve JEDI issues, because all human relationships, including those with the biophysical environment, or more-than-human relationships, are embedded in structures of power that are systematic, durable, and pervasive (Braun 2004; Gagnon et al. 2022). These structures of power represent a continually evolving enactment of the colonialist, extractivist systems involving centuries of systematic oppression based on racialized and ethnic categorizations, gender and sexual identities, and other ascribed attributes used to differentiate among humans and categorize them in hierarchical social structures (Böhme 1997). These relations of power have sweeping consequences, creating inequitable access to all kinds of opportunities and forms of capital, including the ability to participate in decision making that impacts individual lives, as well as local, state, and national planning, development, and governance (e.g., Brandt et al. 2018).

Research opportunities in the context of JEDI focus on addressing the power differentials between researchers, practitioners, and various types of stakeholders that are caused by systematic oppression based on racialized and ethnic categorizations, gender and sexual identities, and other ascribed attributes. Specifically, to begin to address issues of justice, equity, diversity, and inclusion, engagement work should involve research that aims to understand and respect multiple community preferences for how they are engaged and how they are involved in decision making. This in turn builds capacity for diverse and also marginalized communities to determine how and when they participate in engagement. This vision is distinct from engaging for the purpose of ‘educating’ communities or other stakeholders. As has long been a best practice among many collaboration practitioners, engagement processes must begin by acknowledging that stakeholders and communities are not homogenous entities. This is particularly important for marginalized groups where research must aim to better understand intersectional and multidimensional publics rather than presuming homogeneity within or among groups of participants. There is an urgent need for research that examines processes of consent, how consent operates in different engagement processes (across agencies that engage and particular projects that involve engagement, as well as how it varies for rightsholders as compared to stakeholders), how it influences issues of JEDI in engagement, how consent is linked to data sovereignty (Kukutai and Tayler 2016), and how language shapes processes of participation in engagement (Gagnon et al. 2022). Future research on issues of JEDI in engagement should also pay particular attention to connections to place and variation across how both place and time become meaningful in the context of real lived experiences across diverse groups. Thus, we suggest the following research opportunities:

#### 3.1.1 Research about understanding and respecting community preferences for how they are engaged

This research should acknowledge that preferences for engagement and participation in and with decision making vary across communities, and that communities and stakeholders are more diverse than current literature suggests.

#### 3.1.2 Research that addresses intersectional and multidimensional publics

This research should recognize that stakeholders and communities are not homogenous either within or across communities.

#### 3.1.3 Research that directly addresses relationships to and the meanings of land

This research should recognize the diversity in these relations and meanings and how this diversity may limit who participates and who benefits from participation in engagement processes. In other words, space and place are not just contextual, but are analytically relevant.

#### 3.1.4 Research that directly addresses complexity in senses of time

This research should recognize that temporal attachments to place vary across groups and that divergences in these attachments can create barriers to engagement. In addition, this research should address what priorities are considered temporally urgent and recognize that who gets to decide is itself an issue of power and inequity.

#### 3.1.5 Research on data sovereignty, integration of Indigenous knowledge systems, and Tribal Nations and First Nations engagement and decision making

This research should explore how Indigenous knowledge systems can be privileged in engagement, bring new insight for engagement, and explore how best to provide and/or ensure resources necessary for Tribal and First Nations groups’ participation.

#### 3.1.6 Research on consent

This research should address what consent means and how it is used in different forms of engagement, taking justice, equity, diversity, and inclusion into account.

#### 3.1.7 Research on the changing forms of engagement, including openings for hybrid and digital engagements, considering the COVID-19 pandemic, and their implications for justice, equity, diversity, and inclusion

The changes brought about by COVID-19 have significant, but not fully understood JEDI issues and are likely to have lingering effects.

### 3.2 Theme 2: Ethics

Individuals all make moral and ethical choices in deciding to engage, as well as in making decisions about who and how to engage. These choices are shaped by worldviews, which, if left unexamined, open the possibility of amplifying the uneven distribution of burdens, benefits, and risk among both human and more-than-human stakeholders and further entrenching systemic marginalization of those routinely excluded from participation. Because of this, questions surrounding the ethical implications of stakeholder engagement are essential to the fair and successful enactment of engagement-driven research practice, and in particular ensuring participants do not suffer any harm (Wilmer et al. 2021). Stakeholder engagement allows for people who are affected by the problems that researchers seek to solve, and the practices researchers employ, to have a role in determining what should be researched and how the research should be used. Thus, stakeholder engagement holds promise for opening up a more moral and ethical form of research than traditionally practiced by academics. However, it also opens up a set of ethical and justice challenges related to whose ways of knowing and being are considered, enacted, and respected, while simultaneously exposing stakeholders to a set of risks not associated with traditional research practice. This is to say that engagement processes may lead to risks for humans, as well as landscapes themselves.

More broadly, implicit in all research are underlying philosophical orientations which reflect ontological (what exists) and epistemological (ways of knowing what exists) perspectives. It is in acknowledging these orientations that the ethics of stakeholder engagement unfolds as issues of justice are identified and addressed (e.g., procedural, distributive, recognitional; see Bennett et al. 2019) given the context of research. Central to effective engagement is creating enabling settings that allow individuals to consider and learn about their differences openly and productively (Alter et al. 2017). This requires individuals to acknowledge and show respect for each other’s values, beliefs, and dignity as human beings and the ‘gifts’ of knowledge, experience, and insight each person brings to the discussion (Fischer 2000, 2013). It involves acknowledging and respecting beyond one’s own ontological view of the world and the way it functions and for whom. It also involves acknowledging and respecting differing epistemologies or ways of knowing, seeing value in both expert and practical knowledge and experience. This ‘thick’ engagement approach involves always, as Palmer (2011, p. 38–45) puts it, seeing one-self as the ‘other,’ not as the center or fount of understanding, expertise, and insight. Likewise, it involves re-appraising the anthropocentric view of ‘stakeholder engagement in working landscapes,’ which predominantly centers the people involved with landscapes that serve human needs. A broader ontological understanding of ‘stakeholder’ engagement in working landscapes would help address the injustices of the Anthropocene by considering both social and ecological justices (see Whatmore 2002; Davis et al. 2019). Embracing or not embracing these and other ethical principles and associated behaviors is a moral choice itself in that one is either implicitly or explicitly rejecting or open to understanding and learning from the ontologies, epistemologies, values and beliefs, and humanness of others, while either centering or reflexively examining one’s own perspectives, concerns, and interests.

Acknowledging the need for more moral and ethical stakeholder engagement necessarily opens up a reconsideration of the notion of ‘stakeholder’ and the framing of engagement in ways that achieve social, environmental, and ecological justice. Rather than status quo engagement, engagement approaches that facilitate more diverse, equitable, and inclusive participation must be uncovered and employed. These approaches should recognize and mitigate risks for human and more-than-human actors, bridge differences, and bring new voices and new information to the table and ultimately help to shift and share responsibility, power, and decision making. While the stakeholder engagement literature has begun to grapple with these issues, through our working group process we identified several underexplored ethics and justice research questions and gaps that should be addressed to provide a pathway toward achieving more moral, ethical, and just engagement. These research opportunities are summarized below:

#### 3.2.1 Research on the dynamics of ontological and epistemic politics in particular situations, with particular issues, and in general

This research is tasked with recognizing how and why we view the world the way we do (philosophical orientation/positionality) and in so doing makes us aware of our ethical approach to research (issues of justice come up here: who is included, who is left out, etc.)

#### 3.2.2 Research on the relationship between power, politics, and risks of engagement including knowledge co-production

This research should examine how power/political dynamics can lead to situations in which more vulnerable groups/actors end up shouldering most of the risks of engaging and collaborating.

#### 3.2.3 Research aimed at integrating different ways of knowing while co-producing knowledge that is actionable and that contributes to effective and legitimate solutions and the transformation of society

This research should identify factors that enable or constrain achieving these aims and under what conditions and explore how these types of impacts can be assessed.

#### 3.2.4 Research to understand how social values and engagement processes are co-produced, how they co-evolve, and how this co-influenced relationship shapes outcomes

Implicit in all engagement activities are (participants’ and researchers’) held values. This research should seek to understand the dynamics between values and engagement and how this entanglement influences process and outcomes.

#### 3.2.5 Research to determine under what conditions engagement makes issues worse

This research should incorporate consideration for who decides when to engage (or not) and on what topics and seek to explore situations when participation is not seen as necessary or when engagement itself can lead to loss of power.

#### 3.2.6 Research using non-anthropocentric (or multi-ontological) understandings of ‘stakeholders’

Posthumanism, for example, decenters humans and asserts equal rights to more-than-humans, thereby broadening the inclusiveness, positionalities, and standpoints linked with the term ‘stakeholder.’ This research should consider nature as a stakeholder in engagement activities, especially when addressing environmental issues and/or striving for ecological justice.

#### 3.2.7 Research that reconceptualizes the term ‘working landscapes’ by acknowledging multiple ways of knowing landscapes

This research should consider by whom/what the landscape is working and for whom/what it is working. It should also consider the ethical and more-than-human/non-anthropocentric implications of the term.

### 3.3 Theme 3: Research and practice

This theme focuses on research opportunities that aim to span boundaries and build strong and durable connections between researchers and diverse practitioners. Advancing scholarship and practice of engagement demands transdisciplinary collaborations that involve experts across many disciplines, within and beyond academia, and throughout the entire research process (Gibbons et al. 2000; Prokopy et al. 2015; Flint et al. 2019). While working across academic and practical knowledge has proven essential to addressing socio-ecological challenges, there are difficulties in building and maintaining relationships across fields and organizations (Burbach et al. this issue). Along with relational challenges, the reward and incentive system of the academy, the value placed on traditional academic output, and lack of experience and training, among other barriers, prevent research from being widely accepted, adopted, or supported by communities outside academia (Jacobson et al. 2004, p. 249). These barriers must be overcome by universities, governments, and other organizations that want to have successful translational research at local, national, and international levels.

Bridging the gap between research and practice requires research that tackles the existing barriers to effective partnerships (Cash et al. 2006). Barriers to spanning practice–research boundaries to address complex socio-ecological challenges in working landscapes exist within academic, government, and practitioner communities working in agricultural, forestry, coastal, land use, water quality and quantity, and other natural resource contexts (Sabatier et al. 2005). Within universities, barriers include limited incentives or funding for academics to work closely with external partners, emphasis on peer-reviewed publications versus applied research focused on addressing practical versus basic science problems, and the slower project timescales of academic work relative to practitioner problem-solving needs (Anderegg 2010; Arnott et al. 2020; Dilling and Lemos 2011). Within practitioner communities, barriers to engaging scholars and scholarly research, including across disciplines, include academic terminology and language used within and beyond research settings that is inaccessible for non-academic audiences (Xiang 2020), training in interpreting and applying scientific research products (Jacobson et al. 2004), and cost to access research published in peer-reviewed journals behind paywalls. Within research groups, there is a lack of knowledge of theoretical framework approaches to successfully establish research socio-ecological practice (Xiang, 2017, 2021), and practice in how to leverage knowledge brokers, boundary organizations, policymakers while co-producing and/or using integrative research strategies to answer socio-ecological problems (Mach et al. 2020; Cooke et al., 2021). Within government agencies and not-for-profit organizations with responsibilities for managing natural resources, barriers for connecting research and practice include limitations on time/staffing/person-hours, insufficient funding for project support, potential mismatch between agency mission and on-the-ground needs, and costs of accessing relevant scientific research. In the U.S., there are programs and specific roles within government agencies that try to bridge gaps between research and practice, but given the challenge of this work, more investigation into and dialogue about how to better span these boundaries is needed. Research planning that brings academic, policymaker, stakeholder, and practitioner knowledge together to define goals, research questions, usable products, and timelines that serve all involved may help overcome these durable barriers (Xiang, 2019; Forester, 2020). Opportunities under the research and practice theme are summarized below:

#### 3.3.1 Research aimed at understanding best practices for supporting engagement work within universities

This research should explore ways to move beyond talk and see more engagement work in practice, including examining the incentive structure in place to reward diverse faculty and Extension professionals for doing engagement work. It should also recognize the importance of supporting and nurturing long-term relationship building and the time and effort required and see that as critical for building a foundation for effective engaged research.

#### 3.3.2 Research on the effective use of language to overcome barriers to communication, access, and mutual understanding of frames and perspectives

This research should address how standard academic communication norms (jargon, publication primarily in peer-reviewed journals) can limit access and suggest alternative modes of communication and methods for negotiating language and frames in support of improved engagements and implementation of solutions.

#### 3.3.3 Research to understand barriers to accomplishing ‘engagement’ among researchers, practitioners, community stakeholders, and research and practice

This includes access to research, journals, and scientific literature among practitioners and community stakeholders. This research should help understand stumbling blocks and identify ways that people have worked around them.

#### 3.3.4 Research on the lack of long-term engagement with stakeholders

This research should focus on building learning communities and recognize that active relationships with stakeholders define the success of the implementation and adoption of future research projects.

#### 3.3.5 Research on including decision makers / sponsors / convenors as key stakeholders to help understand the learning connection between research and practice (a) within universities and (b) with policymakers and other decision makers outside academia

This research should recognize that these are the people tasked with funding, convening, and implementing engagement and products resulting from engagement processes within and outside academia.

### 3.4 Theme 4: Context

This theme focused on contextual factors that shape stakeholder engagement processes and outcomes. We define the engagement context as preexisting conditions internal or external to stakeholder participants that provide more conducive or challenging circumstances for achieving desired ends through engagement (Eaton et al. 2021, p. 1115–1117). External factors are structural forces extending beyond the immediate influence of participants, for example, funding agency restrictions and conceptions of research, as well as legal and regulatory frameworks. External factors relevant for university researcher and practitioner involvement with stakeholder engagement include university and community expectations and institutions, such as tenure and promotion requirements, research ethics and internal review boards, and community councils and review boards. These structures create the rules of engagement, and structure bounds of what can be accomplished through collaborative approaches. For example, institutional norms and requirements can create tensions among researcher and community stakeholder values and expectations (Blee and Currier 2011; Vanloqueren and Baret 2009). Internal factors include the worldviews and social positions of individual engagement participants that shape how a stakeholder engagement process unfolds. This includes researcher, practitioner, and stakeholder characteristics, norms, values, collaborative skill sets, and understandings and worldviews evident at the outset of an engagement process.

Asking questions about engagement through a lens of contextual factors includes asking how terms such as ‘stakeholder,”\ ‘community,’ and ‘engagement’ are defined and by whom, and which and how stakeholders and communities are engaged. This is important because how researchers, practitioners, and participants define and conceive the term stakeholder and relationships with stakeholders impact the rationales and contexts of engagement (Bendtsen et al. 2021). Likewise, viewing engagement through a contextual lens invites reexamining how the term community is imagined and responded to through community engagement processes. This can start, for example, by exploring a community’s patterns and relationships to move toward better understanding and integration of diverse sociocultural perspectives and knowledge evident within a community, or ‘deep engagement’ (Crick 2012, p. 52). Examining how varying conceptualizations and definitions for key terms at the heart of stakeholder engagement influence power dynamics (and meta-power, i.e., the power to establish, reform, and transform systems, institutions, and hierarchies (Baumgartner et al. 1975, p. 1)) supports the renegotiation of power structures shaping stakeholder engagement processes and outcomes. Research opportunities under the context theme are summarized below:

#### 3.4.1 Research to examine how power and politics at the local, regional, and national levels, and within stakeholder communities, affect and are affected by stakeholder engagement

This research should acknowledge that reexamining power and meta-power dynamics is needed for the renegotiation of power toward democratic engagement practices and relationships, revision of communication norms toward improved understanding, increases credibility of the research process, and improved utility to and uptake of outcomes by stakeholders.

#### 3.4.2 Research to identify how researchers can bridge distances and divides among institutions and communities

This research should understand and acknowledge differences between campuses (and other institutions) and communities, specifically concerning norms, practices, and epistemologies. This understanding enables strategy development for overcoming barriers, development of shared frames, and acknowledging and including community voice in the engagement process.

#### 3.4.3 Research on how the diversity of stakeholder roles and perspectives, including varied definitions of ‘stakeholder,’ impact engagement

This research should acknowledge that understanding the diversity of stakeholders, their roles in the research process, and the diversity of perspectives they hold supports a more inclusive approach to stakeholder engagement and a more nuanced understanding of a community’s multiple voices and improves communication within the process and communication of outcomes.

#### 3.4.4 Research on co-producing understanding for ‘deep engagement’ across disciplines and constituents

Deep engagement involves a commitment by university and community members to co-learn in long-term, substantive, and purposeful relationships that yield constructive results for communities. This research should work to co-produce a shared understanding of the concept of deep engagement to equip researchers, practitioners, and stakeholders across fields and experiences with the means to investigate how to engage meaningfully and with more diverse groups, including those who might disagree with the purpose, scope, or ideology of a project.

### 3.5 Theme 5: Process

An important point of emphasis in recent socio-ecological stakeholder engagement literature has been to understand if, when, and how different approaches to and design features of stakeholder engagement processes affect social and environmental outcomes (Eaton et al. 2021). This work points to the importance of attending to who initiates engagement and how research is communicated (Reed et al. 2018), how decision-making power and inclusion are addressed (Jones et al. 2014), how different processes do or do not build trust and shared understanding (Ansell and Gash 2008), and more. These frameworks are good at illustrating how processes should be designed, but they lack explicit linkages between modes of participation and social–ecological outcomes (Feist et al. 2020).

Here, we identify several research opportunities that focus on the design and processes of stakeholder engagement, along with systematic tracking of how these link to socio-ecological outcomes. Guidance is also needed on how to best communicate research recommendations. We conceptualize engagement through modes (the approaches and methods used to engage) and modalities (how stakeholder knowledge is invited and legitimized, e.g., ranging from communication to knowledge co-production/empowerment) of engagement. ^5^ A better understanding of the ways in which modes and modalities are connected to engagement approaches can help inform stakeholder processes to achieve just, equitable, inclusive, and resilient outcomes. Research opportunities under the process theme are summarized below:

#### 3.5.1 Research analyzing modes and modalities of engagement toward developing a consistent conceptual typology

This research will improve research reporting to enable comparisons of different engagement modes and modalities, enabling cross-case comparisons of approaches, and will also allow for better case-specific assessment of which stakeholders were engaged and outcomes accomplished.

#### 3.5.2 Research to synthesize and integrate engagement case studies to identify broader patterns in linking engagement modes and outcomes

This type of synthesis is required to provide an empirical basis for identifying which modes of engagement are associated with various outcomes, so facilitators can select the modes most likely to attain their desired outcomes.

#### 3.5.3 Research analyzing engagement processes across contexts to identify how local history and context shape modes and outcomes

Comparing and synthesizing studies conducted in different contexts can help explain how institutional, political, and sociocultural context and local history interact to shape which modes and modalities are selected and how these are linked to specific outcomes.

#### 3.5.4 Longitudinal research to capture dynamics between engagement modes and outcomes over time

These efforts can explore whether outcomes from short-term or one-off engagement processes persist over time. Additionally, longitudinal research allows for exploration of which intermediate outcomes (like trust, buy-in, ownership, and commitment among participating stakeholders) are linked to more long-term outcomes (like changes in environmental conditions resulting from engagement processes).

#### 3.5.5 Research on how engagement process design affects types and levels of participation

This research includes identifying best practices to determine the appropriate level of participation for different stakeholders at different phases of an engagement process. It also includes processes to determine when stakeholder engagement may be detrimental to the desired outcomes or to stakeholders themselves. These endeavors require robust information about how selected modes and modalities affect the types and levels of participation from different social groups. Relevant topics include the recruitment/invitation process (who is invited, who does the inviting), design of engagement methods (who can participate, in what capacity, and with what resources), and ways to address the impact of past engagement processes on current willingness of stakeholders to participate.

#### 3.5.6 Research on how to best communicate recommendations to practitioners

This research requires clarity surrounding whose outcomes are researched and recognition that certain communication approaches will privilege different potential audiences. Emphasis should be placed on open access, public sphere reports and peer-reviewed research articles accessible to diverse actors.

### 3.6 Theme 6: Outcomes and measurement

There is a need for research that focuses on social, behavioral, and environmental outcomes of stakeholder engagement processes and how to measure these outcomes. A principal goal of stakeholder engagement in working landscapes is to enable desired change in complex socio-ecological challenges. This includes both on-the-ground environmental change (e.g., improved air, soil, and water quality), as well as cognitive and behavioral change among individuals and groups charged with managing natural resources (Muro and Jeffrey 2012). While stakeholder engagement processes have the potential for enabling such change, there is not yet a strong evidence base for whether and how these processes lead to a range of more positive or negative outcomes (Gerlak et al. 2018; 2019). To illuminate pathways to beneficial outcomes and achieve lasting environmental and social goals, a better understanding of the linkages across contextual circumstances, process factors, and a range of outcomes is needed (Schusler et al. 2003; Singletary and Steele 2020).

We define outcomes as evidence of change attributable to stakeholder engagement in three domains: social, behavioral, and environmental. Social outcomes include changes at the individual and group levels. These include change in how people define and understand the problem or opportunity at hand, change in how people relate to one another, change in social norms that condition how people behave, change in underlying beliefs, and change in the level of skills or collaborative capacities individuals possess (Reed et al. 2010; Emerson and Smutko 2011; Muro and Jeffrey 2012). Behavioral outcomes relate to implementation of plans developed through engagement processes, project design and coordination, land management behaviors, and other tangible outputs of stakeholder engagement projects (Koontz and Thomas 2006). Environmental outcomes include local- to regionalscale outcomes with evidence of changes in biophysical conditions (Eaton et al. 2021). Research opportunities under the outcomes theme are summarized below:

#### 3.6.1 Research on relationships between engagement process design features and social learning outcomes

Research linking process design features and social learning outcomes remain underdeveloped. Causal linkages are often assumed rather than demonstrated empirically. This research is needed to better understand how to design effective approaches to engagement that foster social learning. Research here presents an opportunity for practitioners and researchers to jointly design and undertake research to incorporate new scientific knowledge into practice.

#### 3.6.2 Research on relationships between social learning outcomes and behavior change

Linkages across social learning outcomes and demonstrated change in behavior, at individual and group levels, remain tenuous. This research should aim to identify what evidence for linkages exist (if any), what types of learning are effective for promoting desirable change in human behavior, and how best to measure these relationships.

#### 3.6.3 Research on relationships between behavioral change and socio-environmental outcomes

Numerous challenges exist for linking behavior change resulting from stakeholder engagement outcomes and improvement in socio-environmental conditions. This research should identify practical, useful, and timely means for assessing these linkages.

#### 3.6.4 Research methods for conducting stakeholder engagement research that serves the dual purpose of scholarly and practical aims

Research *on* stakeholder engagement *is* engagement, although this often goes unacknowledged in the literature. Better methods for conducting research and evaluation that at once pursue both scholarly goals for empirically linking engagement contexts, processes, and outcomes, and practical goals for supporting engagement efforts and their objectives (e.g., policy change, resource management goals, etc.) are needed. This is a place where researchers and practitioners could co-design a process to achieve both goals.

#### 3.6.5 Research on contextual factors supporting desirable social, behavioral, environmental outcomes through stakeholder engagement

Research linking contextual factors or enabling environments conducive for desirable social, behavioral, or socio-environmental outcomes remains scant. This research should review what we know about the role of contextual factors in shaping outcomes and describe next steps for improving knowledge.

## 4 Synergies across themes

While the workshop process resulted in the above six themes that shaped the working group process and the research agenda described here, these themes are clearly overlapping and cross-cutting. For example, issues of justice, equity, diversity, and inclusion are deeply tied to questions of ethics and links between research and practice. JEDI issues are overarching, and they cannot be treated as an add-on to existing project frameworks, processes, or decisions. One way this workshop process reflected its overarching nature was by including the JEDI workshop described above, and by facilitating context, process, and outcomes working groups’ dialogue on these concepts through JEDI and ethics lenses.

Readers should not see the themes above as mutually exclusive. Instead, many of these themes are relevant to all forms of engagement. For example, engagement always involves context, process, and outcomes. However, holding our collective attention on each distinct component can help build a more comprehensive view of the ‘inner workings of engagement’ (Feist et al. 2020)—including the qualities of relationships across contextual and process-related circumstances and factors, and a range of related outcomes. Moreover, JEDI is an issue that all scholars and practitioners should be actively working on by educating themselves, unlearning elements of the education already received in a system dominated by inequity and considering how their work either perpetuates or works to address systems of oppression. These considerations are relevant in all forms of engagement, not just those explicitly engaging with groups or individuals who are harmed by current systems of inequity.

## 5 Next steps: putting the research agenda to work

This paper describes a co-produced response to needs identified through iterative and facilitated dialogue among researchers, practitioners, students, and other participants for an actionable agenda for stakeholder engagement research and practice. Recent scholarship highlights the need for better understanding of the inner workings of collaboration, including empirical evidence for relationships across contextual and process-related factors and mechanisms that drive a range of social and environmental outcomes. While compelling and important, this scholarly assessment is largely disconnected from perspectives that emphasize practical needs and insights, as well as explicit questions regarding justice, equity, diversity, and inclusion, and ethical dimensions of stakeholder engagement. Our co-production process provides a novel response for bridging this lacuna. We intentionally welcomed multidisciplinary participants to share scholarly, practical, experiential, and personal knowledge of and insights into stakeholder engagement. We did this through facilitated activities that catalyzed dialogue, welcomed unanticipated topics and themes, and built a shared vocabulary across the disciplines and practices involved. We sustained this dialogue with formal and informal activities including virtual workshops, supporting working group activities, and other discussions as needed throughout a yearlong process. In describing this research agenda here, we invite critical perspectives that seek to add to or modify our agenda, as well as build new collaborations to pursue these opportunities.

## Supplementary Material

Supplement1

## Figures and Tables

**Table 1:** How participants characterize their engagement work

	Most of my time	Some of my time	Little or none of my time	Total

I conduct academic research on or related to engagement	41.4% (n = 53)	46.1% (n = 59)	12.5% (n = 16)	128
I facilitate engagement as a practitioner	23.4% (n = 30)	39.8% (n = 51)	36.7% (n = 47)	128

## Footnotes

1 These workshops were supported by the Agriculture and Food Research Initiative (AFRI) Advancing scholarship and practice of stakeholder engagement in working landscapes grant no. 2020–01551 project accession no. 1023309 from the USDA National Institute of Food and Agriculture.

2 See the workshop report: https://scholarsphere.psu.edu/resources/d6066f7e-045c-41f7-af69-9cc15e1e81f3

3 The project stopped short of involving participants identifying as ranchers, landowners, producers, community residents, youth, Indigenous community representatives, and other rights holders and stakeholders at the same level of participation as researchers, practitioners, and students, as meeting their goals for engagement was beyond the immediate scope of this project. However, the project did invite stakeholder perspectives (e.g., during the JEDI workshop as described herein).

4 See workshop report for comprehensive findings.

5 This follows the International Association of Public Participation (IAP2)’s 3 Pillars of Public Participation, specifically the IAP2 Spectrum of Public Participation which can be found here: https://www.iap2.org/page/pillars
