# Supplementary material for: Advancing the scholarship and practice of stakeholder engagement in working landscapes: a co-produced research agenda
Source: Socioecol Pract Res. Author manuscript; Available in PMC 2023 Nov 11. (PMC9651121; doi:10.1007/s42532-022-00132-8)
Supplement: Supplement1 [file NIHMS1857583-supplement-Supplement1.pdf]

Appendix A: Participant survey responses

Advancing Scholarship and Practice of Stakeholder Engagement in Working Landscapes Workshop Series

123 workshop participants (October 19, 2020)
118 survey respondents (October 15, 2020)

Gender and ethnic/racial identity of participants (n=118)

Survey questions: “What is your gender identity?” and “What is your race or ethnicity?” Open-ended response

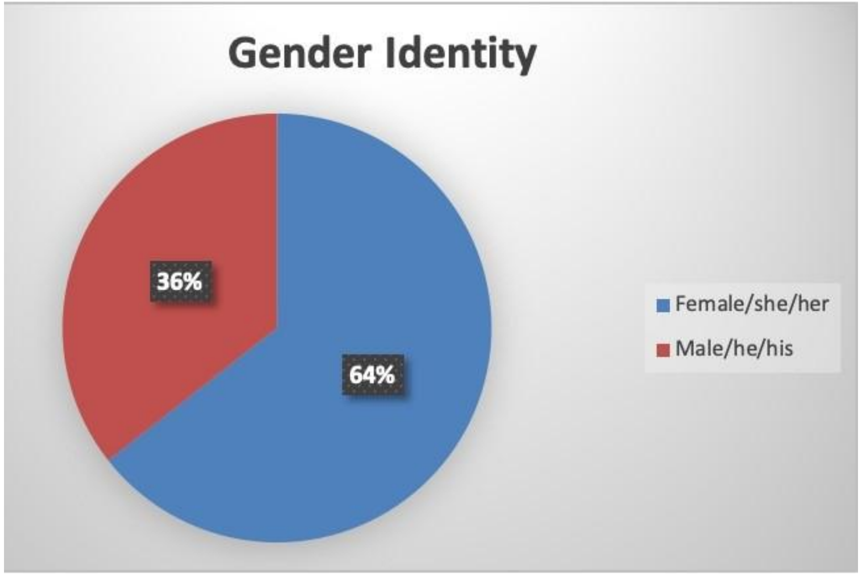

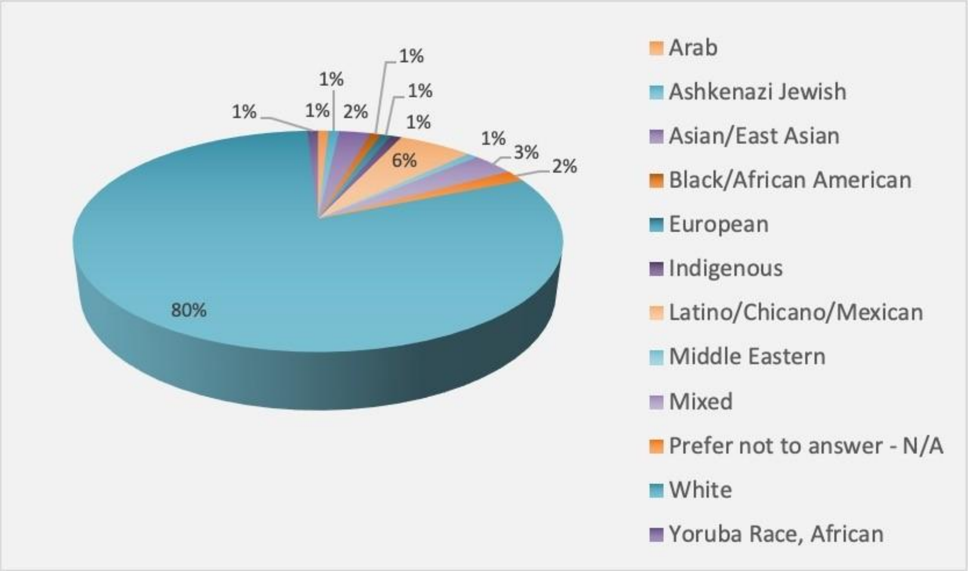

Countries where participants do engaged work

Survey question: “Where do you primarily do your research/work on stakeholder engagement?” Text box answer to “Country”

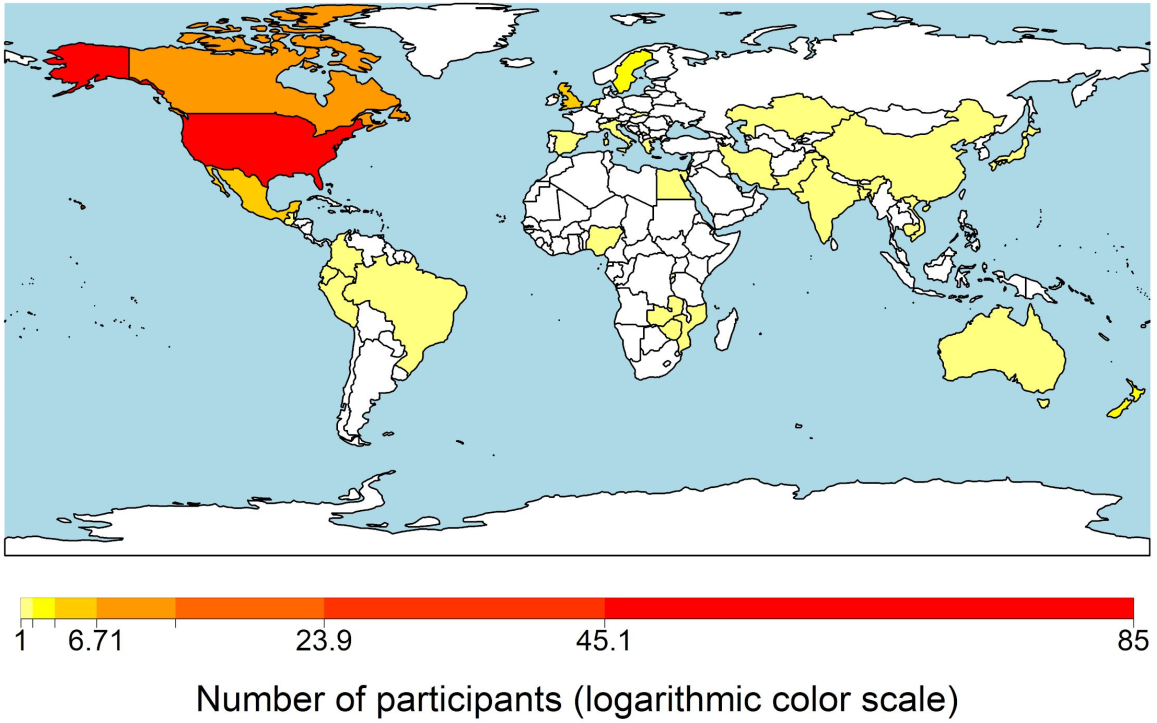

Country data

- USA = 85
- Canada = 8
- United Kingdom = 5
- Mexico = 5
- New Zealand = 3
- Sweden = 2
- The Netherlands = 2
- others in yellow= 1
- white = 0 people

Sample information

- 33 countries
- 118 respondents
- 136 people-countries

States/regions where participants do engaged work

Survey question: “Where do you primarily do your research/work on stakeholder engagement?” Text box answer to “Region” or “State”

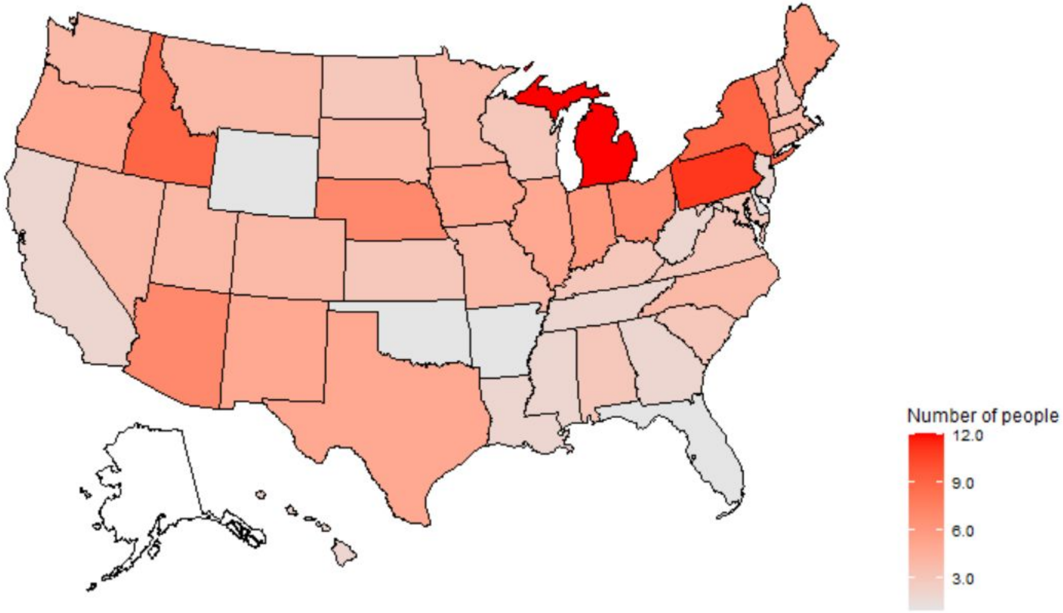

<u>US States data</u>

- Michigan = 12
- Pennsylvania = 11
- Idaho, New York = 9
- Nebraska, Arizona, Ohio = 7
- Maine, Indiana = 6
- Iowa, Illinois, New Mexico, Oregon, Texas, Vermont = 5
- others < 5

<u>Sample information</u>

- 49 states + Puerto Rico (2) and Pacific Islands (1) (not shown)
- 85 respondents
- 209 people-states
- Five people work nationwide (not shown)

Landscapes and settings where participants work (n=118)

Survey question: "In which types of working landscapes/settings are you researching or practicing engagement? (check all that apply)"

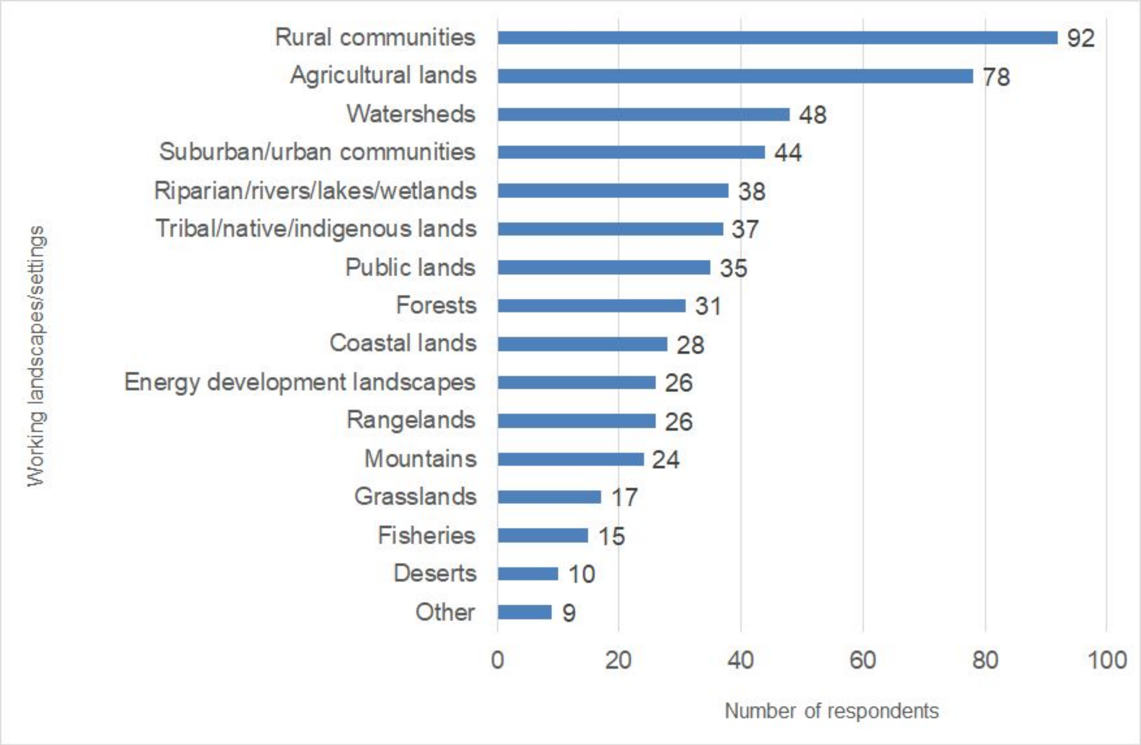

Respondents work in many different landscapes/settings:

8 respondents: 1
37 respondents: 2-3
34 respondents: 4-5
30 respondents: 6-9
9 respondents: 10 or more

Other categories include:

Agricultural Reserve lands
Cities, gated compounds
Climate change adaptation and development
Food, Energy, Water stakeholders
Invasive species (...in any type of landscape)
Sagebrush steppe landscapes
Snow-fed rivers in arid lands
Urban waterfronts
Working waterfronts

Topics/issues participants engage with (n=118)

Survey question: “Which topics/issues have been the focus of your engagement practice and/or research? (check all that apply)”

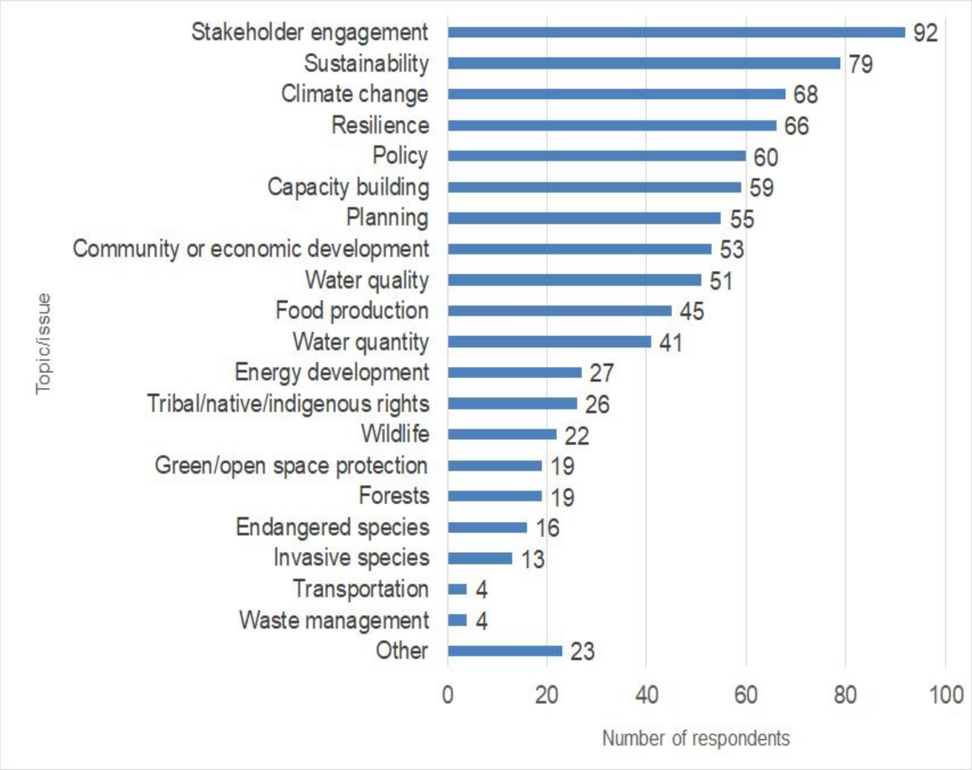

Other categories include:

Arts science dialogue
Collaborative natural resource management
Collaborative/participatory design, methods, evaluation of
Cultural identity/historic preservation
Ecological monitoring in general
Energy development impacts to school/community viability
Farmland preservation
Flooding
Food security
Gender dynamics of labor on working lands
Habitat restoration
Health (general)
Intersection between agriculture and health
Justice, education, media and communication
Land ownership and wealth building
Land use and land use change
Landscape aesthetics
Livelihoods and access equity to environment/natural resources
Monitoring and management
Natural Hazards
Non-food agricultural production…
Participation in decision-making
Racial equity and decoloniality
STEM engagement
Tourism and recreation
Transboundary aquifers
User conflict
Wildfire
Wildland Urban Interface behavior

Respondents work on many different topics/issues:

2 respondents: 1
15 respondents: 2-3
27 respondents: 4-5
53 respondents: 6-9
21 respondents: 10 or more

Who participants are engaging and who participants would like to engage more

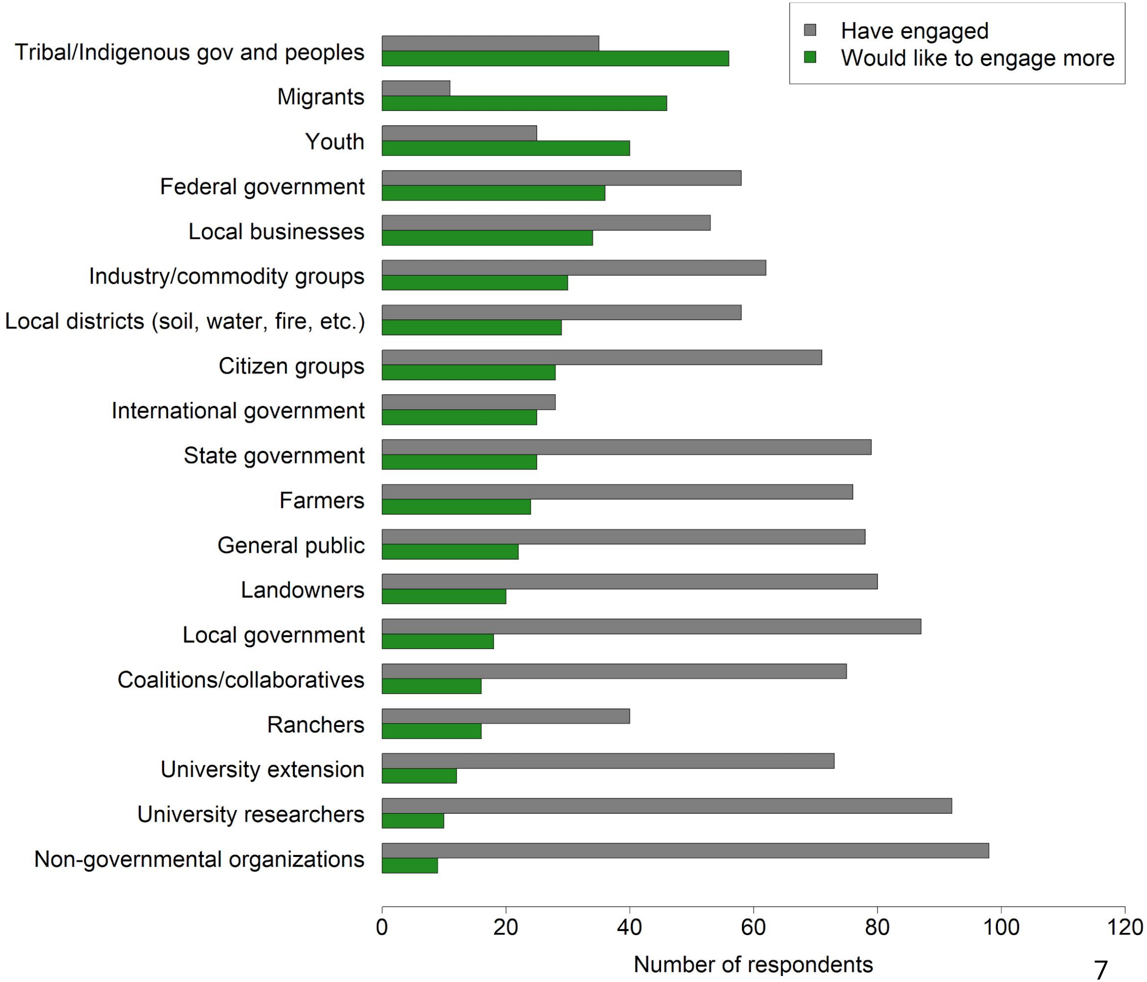

Survey question: “In your research or engagement work, please indicate the level of engagement from each of the following types of working lands stakeholders.” Response options were: “Have engaged often,” “Have engaged somewhat,” “Would like to engage more,” and “N/A.” The first two response options were combined for the figure shown here.
